# Assessments of early patellofemoral joint osteoarthritis features after anterior cruciate ligament reconstruction: a cross-sectional study

**DOI:** 10.1186/s12891-023-06639-9

**Published:** 2023-06-22

**Authors:** Michael Tim-yun Ong, Gene Chi-wai Man, Xin He, Mingqian Yu, Lawrence Chun-man Lau, Jihong Qiu, Qianwen Wang, Jeremy Ho-pak Liu, Ben Chi-yin Choi, Jonathan Patrick Ng, Patrick Shu-hang Yung

**Affiliations:** 1 Department of Orthopaedics and Traumatology, Faculty of Medicine, the Chinese University of Hong Kong, 74029 Hong Kong SAR, China; 2 Lui Che Woo Clinical Science Building, Prince of Wales Hospital, Shatin, Hong Kong SAR China; 3 Department of Orthopaedics and Traumatology, Prince of Wales Hospital, Hong Kong SAR, China

**Keywords:** Patellofemoral joint, Osteoarthritis, Anterior cruciate ligament reconstruction, Quadriceps, Pain

## Abstract

**Background:**

Persistent anterior knee pain and subsequent patellofemoral joint (PFJ) osteoarthritis (OA) are common symptoms after anterior cruciate ligament reconstruction (ACLR). Quadriceps weakness and atrophy is also common after ACLR. This can be contributed by arthrogenic muscle inhibition and disuse, caused by joint swelling, pain, and inflammation after surgery. With quadriceps atrophy and weakness are associated with PFJ pain, this can cause further disuse exacerbating muscle atrophy. Herein, this study aims to identify early changes in musculoskeletal, functional and quality of health parameters for knee OA after 5 years of ACLR.

**Methods:**

Patients treated with arthroscopically assisted single-bundle ACLR using hamstrings graft for more than 5 years were identified and recruited from our clinic registry. Those with persistent anterior knee pain were invited back for our follow-up study. For all participants, basic clinical demography and standard knee X-ray were taken. Likewise, clinical history, symptomatology, and physical examination were performed to confirm isolated PFJ pain. Outcome measures including leg quadriceps quality using ultrasound, functional performance using pressure mat and pain using self-reported questionnaires (KOOS, Kujala and IKDC) were assessed. Interobserver reproducibility was assessed by two reviewers.

**Results:**

A total of 19 patients with unilateral injury who had undergone ACLR 5-years ago with persistent anterior knee pain participated in this present study. Toward the muscle quality, thinner vastus medialis and more stiffness in vastus lateralis were found in post-ACLR knees (p < 0.05). Functionally, patients with more anterior knee pain tended to shift more of their body weight towards the non-injured limb with increasing knee flexion. In accordance, rectus femoris muscle stiffness in the ACLR knee was significantly correlated with pain (p < 0.05).

**Conclusion:**

In this study, it was found that patients having higher degree of anterior knee pain were associated with higher *vastus medialis* muscle stiffness and thinner *vastus lateralis* muscle thickness. Similarly, patients with more anterior knee pain tended to shift more of their body weight towards the non-injured limb leading to an abnormal PFJ loading. Taken together, this current study helped to indicate that persistent quadriceps muscle weakness is potential contributing factor to the early development of PFJ pain.

## Background

Anterior cruciate ligament (ACL) injury is one of the most common sport injuries among young patient that requires surgical reconstruction [1, 2]. Affected individuals suffer both time loss from sport and an increased risk of premature osteoarthritis. Anterior knee pain occurs in 30–50% of patients 1–2 years following anterior cruciate ligament re-construction (ACLR) and approximately half of all ACLR patients suffer from radio-graphic patellofemoral (PFJ) osteoarthritis (OA) 10 years after ACLR [3]. However, the site of disease initiation and the sequence of degenerative process in subchondral bone and cartilage remains to be elucidated.

Since ACL injury is most common in active young adults, people who develop OA after ACL injury are typically young or middle aged, and can experience prolonged disability with few effective treatment options [4, 5]. This has been shown to lead to the difficulties on participating in work and increase in psychological distress and quality of life (QoL) impairment [6, 7]. Moreover, those living with knee symptoms and OA for more than five years after ACL injury has been shown to develop mentally distress on the lack of confidence in the knee to do exercise and to participate in desired recreational activities [6]. Most often, the patient would undergo post-operative rehabilitation after ACLR. The rehabilitation aims to help patients to regain mobility and strength, with an ultimate target of return-to-play (RTP) by 12 months post-operatively. Despite well-designed rehabilitation programs, it has been shown that up to 35% of patients failed to return to their level of play before ACL injury [8]. In addition, they are 6 times more likely to sustain further ACL injury within 24 months after ACLR when compared with the healthy population [9].

The strength of the quadriceps muscle is one of the key determinants for a patient succeeding in RTP after ACLR. While muscle size is a crucial factor for muscle strength, both quadriceps atrophy and weakness are common sequelae after ACL injury and ACLR [10, 11]. This can be contributed by arthrogenic muscle inhibition and disuse, caused by joint swelling, pain, and inflammation after surgery. Quadriceps atrophy and weakness are associated with PFJ pain and in turn, can cause further disuse exacerbating muscle atrophy. Quadriceps-strengthening exercises have been shown to be effective to improve symptoms of PFJ OA and greater quadriceps strength after ACLR have been shown to be associated with less severe patellar cartilage damage. Although such exercise rehabilitation program can aid in recovering quadriceps muscle mass, some patients respond poorly and fail to regain muscle mass. Quadriceps muscle atrophy can persist beyond the completion of the rehabilitation program in almost half the patients and the reason behind this is still unknown. If we can identify pre-arthritic changes in PFJ before the development of OA, it may be possible to provide alternative rehabilitation program to improve the quadriceps function and halt the development of OA.

Toward the evaluation of the results after treatment, it is essential to evaluate the QoL, in addition to functionality by means of the clinical assessments. This fundamentally refers to the influence of a person’s health status on their perceived well-being and life quality. Patient-reported outcomes are commonly used to assess Health-related Quality of Life (HR-QoL) questionnaires. However, the long-term effects of associated lesions on the ACL injury remains controversial. In a retrospective cohort study of 225 consecutive patients admitted for physical therapy with ACL, it showed that male gender and sports as the cause of the ACL lesion were factors significantly associated to better QoL at the end of follow-up [12]. This may facilitate targeted strategies to incorporate satisfying activities that would be essential to improve QoL in symptomatic people after ACL reconstruction. Nevertheless, the temporal relationship between meniscal and other concomitant injuries, OA development, and HRQoL after ACLR is not well understood and requires further investigation.

Herein, the purpose of this exploratory study aims to identify early changes in musculoskeletal, functional and quality of health parameters for knee OA 5 years after ACLR for its contribution toward the development of PFJ pain. We hypothesized that the knee undergone ACLR would develop persistent quadriceps muscle atrophy for developing PFJ pain to lower its activities and quality of life.

## Methods

### Ethics Statement

This study complied with the Declaration of Helsinki after obtaining approval from the institutional Clinical Research Ethics Committee (CREC reference number: 2018.604). All subjects were provided with written informed consent prior to joining this study. All details that might disclose the identity of the subjects under this study were omitted.

### Patients and Procedure

Patients with arthroscopically assisted single-bundle ACLR using hamstrings graft for more than 5 years were identified and reviewed from our clinic registry. Those with persistent anterior knee pain were invited back for our follow-up study. For all participants, basic clinical demography and standard knee X-ray were taken. Likewise, clinical history, symptomatology, and physical examination were performed to confirm isolated patellofemoral joint pain. Exclusion criteria include those with ab-normal lower limb alignment &/or knee morphology, concomitant ligament injuries other than ACL, referred pain and generalized ligamentous laxity.

The data from previous studies were used to estimate the sample size for detecting changes in bone stress injures of the patella between individuals with and without patellofemoral pain [13, 14]. With 95% power and α-level of 0.05, 10 individuals would be needed to detect a significant difference between injured and uninjured knees (G*Power software version 3.1.9.5, Germany).

### Muscle thickness and shear modulus measurements

An Aixplorer ultrasound scanner (version 6.0; Supersonic Imagine, Aix-en-Provence, France) coupled with a linear transducer array (2–10 MHz; SuperLinear 10 − 2; Vermon, Tours, France) was used for quadriceps muscle thickness and stiffness measurement [15]. Participants laid supine on a treatment table for the assessment. A measuring tape was used to locate *vastus medialis* (VM), *vastus lateralis* (VL), *rectus femoris* (RF) and the patella by palpation, consequently marked with a pen for reference. For consistency and ease of comparison across patients, the locations measured and labelled as the three muscle groups are as followed. Firstly, RF and VI were marked at 1/2 of the distance from the anterior superior iliac spine (ASIS) to the superior pole of the patella. Next, VM was located at 1/5 of the distance away from the midpoint of the medial patella border to the ASIS. Thirdly, VL was then noted at 1/3 of the distance from the midpoint of the lateral patella border to the ASIS. With these markings, the transducer probe was aligned in the transverse plane and moved along the entire muscle bundle to capture a view of the VM, VL, VI, and RF [16]. The operator would position the probe into the sagittal plane to measure muscle thickness upon the marked anatomical points. Additionally, minimal pressure was applied on the limb to prevent the deformation of the muscle. In brief, the result would be reported from the average of three repeated measurements.

To measure the muscle stiffness, the shear wave elastography mode was used. For each muscle, images were taken after the transducer was held at the measurement site for < 10 s in order to confirm that the shear elastic modulus in the region of interest (ROI) exhibited stable colour distribution. The mean Young’s modulus of a circle with a diameter of 11 cm set near the centre of the ROI was quantified using software installed in the system [17]. The measurements were taken three times for each muscle, and the mean values were used for statistical analysis. As skeletal muscle cannot be assumed to be isotropic, so we report the shear modulus values as the Young’s modulus values divided by 3 [18]. Subjects were asked to stay as relaxed as possible.

### Measurement of plantar forces and pressures

During level barefoot walking, the plantar forces and pressures were recorded using the TekScan MatScan® system (Boston, MA, USA). This system is consisted of a 5-mm thick floor mat, comprising of 2288 resistive sensors, in sampling data at a frequency of 40 Hz (Hz) [19]. Then, a two-step gait initiation protocol was used to obtain foot pressure data [19]. To ensure adequate reliability of pressure data, a total of three trials were recorded [20, 21]. Following data collection, a software (Research Foot version 5.24) was used to determine peak pressures for the whole foot and under seven regions of the foot.

### Health-related QoL questionnaires

#### Knee Injury and Osteoarthritis Outcome score (KOOS)

The KOOS is a knee-specific instrument developed to evaluate functioning in daily living, sport, and knee-related quality of life in patients with knee injuries who are at risk of OA developing. This questionnaire has been validated in several populations to monitor the short- and long-term consequences (i.e., OA) of these injuries. The Chinese version of the KOOS has been validated in patients with different stages of OA [22]. The score consisted of 5 subscales, this includes: Pain (9 items), Symptoms (7 items), Activities of Daily Living (17 items), Sport and Recreation Function (5 items) and knee-related Quality of Life (4 items). Toward each sub-scale, the scores are transformed to a 0 to 100 scale. Those being 0 representing extreme knee problems, while 100 representing no knee problems.

### International knee Documentation Committee subjective knee form

The IKDC is a knee-specific instrument that was developed to measure symptoms, function, and sport activities in patients with a variety of knee problems. This is commonly used in patients who visited orthopaedic sports medicine practices with the preceding injuries. The Chinese version of the IKDC has been validated in patients with a variety of knee-related problems [23]. The questionnaire consists of 18 items and is scored by summing the scores of the individual items (raw score) and then transforming the summed score to a scale ranging from 0 to 100. Higher scores represent lower levels of symptoms and higher levels of function and participation in sports activity.

### Kujala score

The Kujala score is a self-administered questionnaire for patellofemoral disorders that assesses subjective symptoms and functional limitations. The original study group for the score featured patients with subjects with anterior knee pain, patellar subluxation, and patellar dislocation, in addition to a control group. The Chinese version of the Kujala Score has been validated in patients with for patients with patellofemoral pain [24]. The questionnaire involves 13 questions (100 points) that is validated in the context of patellofemoral instability and is often used in the context of TKA.

### Statistical analysis

Descriptive statistics were used to summarize the data. Kolmogorov-Smirnov and Shapiro-Wilk tests were used to determine if variables were normally distributed. The Mann-Whitney U Test and Spearman Correlation Test were used to data analyses. Descriptive statistics were used to summarize the data. All statistical analyses were completed using SPSS statistical software, version 22.0. P values < 0.05 were considered statistically significant.

## Result

### Patient demographic data

A total of 19 patients participated in this study. The average age was 32.0 ± 6.1 years (range 22–42) (Table 1). There were twelve men (63.2%) and seven women (36.8%). The average BMI was 24.7 ± 4.2 kg/m2 (range 16.1–34.1 kg/m2). Arthroscopically assisted single-bundle ACLR using hamstrings graft were performed unilaterally on the right knee of ten patients (52.6%) and left knee in nine patients (47.4%). The average time from surgery was 6.4 ± 1.0 years (range 5–8). There were no signs of growth disturbances at follow-up on the radiographs. The characteristics of the subjects are shown in Table 1.

**Table 1:** Patient demographic information

*Demographic*	
Number of Subjects	19
Age (years) (Range)	32.0 ± 6.1 (22–42)
*Gender*	
Female (%)	7 (36.8%)
*Laterality*	
Left (%)	9 (47.4%)
Right (%)	10 (52.6%)
BMI (kg/m^2^) (Range)	24.7 ± 4.2 (16.7–34.1)
*ACL surgery*	
Age at Surgery (years) (Range)	26.3 ± 5.8 (18–37)
Time from Surgery (years) (Range)	6.4 ± 1.0 (5–8)

Values are presented as mean ± SD (range) or n (%). ACL, anterior cruciate ligament.

### Reliability of the measurements

The ICC of intrarater reliabilities of the shear modulus for VM, RF, VI and VL were 0.963 to 0.981. was indicated excellent reliability (ICC: 0.963 to 0.981). In addition, the ICC of inter-rater reliabilities were 0.622 to 0.891. Overall, the ICC data demonstrated good to excellent reliability among the tested muscles.

### Quadriceps muscle assessments

Quadriceps muscle atrophy and strength deficit were evident in the knee with ACLR, as shown by decreased quadriceps muscle thickness and increased quadriceps muscle stiffness (Table 2). Toward muscle thickness, the difference on *vastus lateralis* was significantly less thick in the ACLR knee (p = 0.018). Toward the ACLR knee, it was found to be more significantly stiffer in the *vastus medialis* muscle (p = 0.048) (Table 2).

**Table 2:** Knee Muscle Quality at Follow-up

	Injured Leg (n = 19)	Non-injured Leg (n = 19)	P-value
*Total Muscle*			
Thickness (cm)	8.2 ± 0.5	8.6 ± 0.4	0.043
Stiffness	29.8 ± 2.5	28.5 ± 2.4	0.116
*Vastus medialis*			
Thickness (cm)	2.3 ± 0.6	2.4 ± 0.6	0.525
Stiffness	9.6 ± 2.2	8.7 ± 1.5	0.048
*Rectus femoris*			
Thickness (cm)	2.1 ± 0.4	2.2 ± 0.3	0.122
Stiffness	11.7 ± 2.7	11.3 ± 2.7	0.450
*Vastus intermedius*			
Thickness (cm)	1.9 ± 0.5	2.0 ± 0.4	0.153
*Vastus lateralis*			
Thickness (cm)	2.1 ± 0.3	2.2 ± 0.4	0.018
Stiffness	9.1 ± 1.6	8.5 ± 1.8	0.413

Values are presented as mean ± SD

### Functional assessments

Toward the function, the assessment on the pressure mat showed that patients with more anterior knee pain tended to shift more of their body weight towards the non-injured knee with increasing knee flexion (Table 3).

**Table 3:** Squat Test at Follow-up

		*Injured Leg*	*Non-Injured Leg*	*P-value*
Standing, N		49.9 ± 5.2	50.1 ± 5.2	0.929
Knee Flexed angle				
30 ^o^		49.4 ± 4.1	51.1 ± 5.3	0.452
60 ^o^		49.4 ± 5.1	50.6 ± 5.1	0.618
90 ^o^		49.2 ± 4.5	50.8 ± 4.5	0.496

Values are presented as mean ± SD

Compared with age-matched healthy controls from a previous literature [25], the results for the KOOS were lower in the categories for symptom, activities of daily living, sport / recreation and quality of life (Table 4). Likewise, the IKDC score and Kujala score were comparatively lowered in our patients when compared with that of healthy controls (Table 4). Toward KOOS pain, the stiffness on rectus femoris was found to be significantly correlated (R = 0.504, p = 0.028), however there was no correlation with the thickness of these muscles (Table 5). Moreover, there was no significant correlation on the uninjured contralateral knee (Table 5).

**Table 4:** KOOS, IKDC, and Kujala at Follow-up

		*Scores at Follow-up*	*Reference Value* ^*a*^
*KOOS*			
Symptom			
Median (range)		89.3 (50–100)	97.2
Mean ± SD		82.1 ± 13.4	92.2
Pain			
Median (range)		95.2 (83–100)	92.9
Mean ± SD		92.9 ± 5.8	88.1
Activities of daily living			
Median (range)		98.5 (78–100)	100.0
Mean ± SD		96.2 ± 5.7	94.7
Sport / recreation			
Median (range)		75.0 (10–100)	93.7
Mean ± SD		76.1 ± 21.1	85.6
Quality of Life			
Median (range)		62.5 (31–81)	90.7
Mean ± SD		61.8 ± 14.0	84.4
*IKDC*			
Median (range)		78.2 (48–97)	
Mean ± SD		76.4 ± 13.6	99.4
*Kujala*			
Median (range)		80.0 (49–91)	
Mean ± SD		77.3 ± 9.9	94.6

IKDC, International Knee Documentation Committee; KOOS, Knee injury and Osteoarthritis Outcome Score

^a^ The reference values relate to healthy, non-operated age-matched controls from previous literatures [24].

**Table 5:** Correlation of Muscle Stiffness with Knee Functions

*Injured Leg*							
*Parameters*	*KOOS - Symptoms*	*KOOS - Pain*	*KOOS - ADL*	*KOOS - Sport*	*KOOS - QoL*	*IKDC Score*	*Kujala Score*
*Total Muscle stiffness*	0.369	0.560*	0.232	0.228	0.480*	0.353	0.243
VM stiffness	0.273	0.308	0.137	0.148	0.390	0.243	0.141
RF stiffness	0.302	0.504*	0.330	0.171	0.363	0.144	0.246
VL stiffness	-0.060	-0.014	0.074	0.019	0.131	0.080	-0.229
***Contralateral Uninjured Leg***							
***Parameters***	***KOOS - Symptoms***	***KOOS - Pain***	***KOOS - ADL***	***KOOS - Sport***	***KOOS - QoL***	***IKDC Score***	***Kujala Score***
*Total Muscle stiffness*	0.017	0.116	-0.115	-0.224	0.155	-0.123	-0.204
VM stiffness	0.009	0.084	-0.109	-0.126	0.216	-0.067	-0.231
RF stiffness	0.110	0.102	-0.033	-0.190	0.054	-0.168	-0.123
VL stiffness	-0.125	0.041	-0.109	-0.114	0.104	0.034	-0.079

Spearman correlation, ** p < 0.01; *p < 0.05

ADL, Activities of daily living; QoL, Quality of Life; VM, *Vastus medialis; RF, Rectus femoris; VI, Vastus intermedius; VL, Vastus lateralis.*

## Discussion

The main finding in this study was that OA changes were more frequently seen on radiographs in patients who underwent ACLR, compared to their uninvolved contralateral knee 3–5 years after surgery. Furthermore, persistent muscle atrophy and poorer knee function were observed in the knee with ACLR.

The changes in muscle characteristic have been shown to play an important role in understanding the effects of longitudinal progression or interventions for knee OA. It should also be pointed out that the joint tissues do not appear to be the only cause of pain and dysfunction in the patient, but that the periarticular muscles are also involved in the clinical presentation of many patients [26, 27]. Often, patients with knee OA typically present with reduced force-generating ability in the quadriceps that can be attributed to muscular atrophy as well as muscular inhibition. These limitations may be attributed to weakness of the quadriceps muscle because quadriceps strength (peak torque generation) is an important determinant of physical function in subjects with knee OA. As shown in current literatures, muscle strength, especially quadriceps, is a major determinant of both performance-based and self-reported physical function in patients associated with knee OA [28]. Although in a recent study performing a secondary analysis of data from a cross-sectional study in patients presenting knee pain OA, it found pain was correlated poorly with the prevalence of periarticular muscles [26]. However, this was limited by a small sample size and the lack of a control group of participants.

Persistent quadriceps dysfunction has been shown to be a primary modifiable factor that contributes to the onset of osteoarthritis [29]. The alterations in kinematics after ACLR has been proposed to be both a contributing factor for posttraumatic osteoarthritis [30], and a risk factor for re-injury [31]. The alteration in knee biomechanics has been quantified by asymmetry in the quadriceps strength in previous studies [31]. As quadriceps strength can be improved with physical therapy, it would be ideal to resolve these persistent strength deficits before patients return to activity. While the rehabilitation techniques have focused on strength restoration after ACLR, there is still no consensus on the criteria for RTP.

Ultrasound imaging was utilized to measure muscle thickness in this study, as opposed to using MRI to measure the muscle volume or cross-sectional area (CSA). Although this may be less accurate, it is more convenient and less labour intensive than the conventional MRI assessment. As literature suggested the CSA measurement is effective in detecting gross quadriceps muscle atrophy but failed to detect changes of individual muscles, such as RF, VL and VM [32]. In addition, the measurement of the CSA at a specific thigh position may be too insensitive to detect subtle changes in the quadriceps muscles in individual patients and the use of ultrasonography measurement of muscle thickness can achieve similar sensitivity to detect quadriceps muscle atrophy using volumetric measurements.

Strengthening exercise is commonly performed as a conservative treatment that patients to relieve musculoskeletal pain symptoms. In particular, quadriceps exercises are widely taught to patients with knee pain [33]. However, there is a controversy regarding the effect of quadriceps muscle exercises to improve knee pain [34, 35]. Some ACLR patients may still develop persistent muscle atrophy despite following established rehabilitation protocol. Normally, the expression of myokines mediates adaptive responses such as muscle hypertrophy in response to exercise. The results of our study can provide a theoretical basis for quadriceps muscle exercises in preventing the progression of PFJ pain for the potential development of knee OA. In addition, a recent article introduces the use of a new ultrasound-guided percutaneous electrolysis and exercise treatment in patellar tendinopathy [36]. The result showed the combination of percutaneous electrolysis and therapeutic exercise may have the potential to improve pain and disability in individuals with patellar tendinopathy. Importantly, this novel treatment is minimal invasive to prevent patients from having to undergo surgery. Yet, further controlled trials of this novel treatment approach are still warranted.

We have previously shown that dysregulation in myokines can lead to ability to regain muscle mass after ACLR, and persistent muscle atrophy. Subsequently, this may cause significant strength deficits and poor functional recovery [37]. Patients with quadriceps muscle atrophy after ACLR demonstrated lower quadriceps strength and significant lower side-to-side muscle thickness ratios. In non-atrophy group, the expression of serum brain-derived neurotrophic factor (BDNF) increase with resistance exercise training (RET), however a decrease was found in the atrophy group. Similarly, altered myokine expressions in several muscle wasting disorders such as diabetes, cancer, and ageing has also been demonstrated in previous studies [38]. Studies have found muscle contraction increase BDNF expression [39].

Unlike previous studies, our current study included the evaluation of the actual thickness of the individual medial and lateral muscles of both knees, rather than the use of muscle measurements from radiological evaluation. However, the current study is not without limitations. Firstly, the current study is limited by a small sample size. In addition, there was also the lack of an interim follow-up with specific assessments (e.g., isokinetic test). Moreover, the results inferred from this study will be more complete if introducing a healthy group as a comparison. In addition, we did not perform an evaluation of the anatomic changes and the tunnel placement. Lastly, the cross-sectional design of the study may lead to selection bias. However, we attempted to reduce bias as much as possible through the exclusion criteria.

### Future directions and clinical implications

Identification of factors that are associated with the development of knee OA after ACL injury is clinically important. Importantly, ACL-reconstructed patients traditionally performed significantly better compared to non-operated patients in both subjective and objective scores. Although ACLR may contribute to restoration of most functional scores, it could be associated with OA progression. In recent years, the importance of rehabilitation programs after ACLR has been highlighted. Evidence suggests that accelerated rehabilitation protocols should be introduce in sequential phases for better outcomes [40]. Such that, phase 1 should typically aims for pain and oedema reduction to regain of range of motion. Whereas, phase 2 would introduces progressive improvement of quadriceps and hamstring strength, prior to improving neuromuscular control [40]. Moreover, when strength and endurance are maximized, specific exercises that aim to fulfil return to sports criteria should then be introduced. Though the importance of maintaining an advanced level of physical activity is critical, attention on improving the mental health of patients is essentially important.

## Conclusion

In this study, it was found that patients having higher degree of anterior knee pain, were associated with lower *vastus lateralis* muscle thickness and higher *vastus medialis* muscle stiffness. The imbalance of muscle mass and stiffness between the *vastus medialis* and *vastus lateralis* could possibly contribute to abnormal patellofemoral joint loading localizing towards the lateral facets resulting in bony sclerosis and osteopenia. Muscle wasting, patellofemoral joint pain and abnormal muscle recruitment during squatting are part of an interlinking viscous cycle which contributed to persistent quadriceps muscle weakness subsequently leading to an abnormal patellofemoral joint loading. With this finding, as future study, we can investigate for the role of additional muscle training or adjunct intervention with an aim to improve the muscle function to improve the symptoms and delay the progression of PFJ OA.

## Data Availability

The datasets used and/or analyzed during the current study are available from the corresponding author on reasonable request.

## List of abbreviations

ACL: Anterior cruciate ligament
ACLR: Anterior cruciate ligament reconstruction
BMI: Body mass index
IKDC: International Knee Documentation Committee
KOOS: Knee Injury and Osteoarthritis Outcome Score
OA: Osteoarthritis
PFJ: Patellofemoral joint
RF: *Rectus femoris*
VI: Va*stus intermedius*
VL: *Vastus lateralis*
VM: *Vastus medialis*
