# Correction: Assessments of early patellofemoral joint osteoarthritis features after anterior cruciate ligament reconstruction: a cross-sectional study

**DOI:** 10.1186/s12891-023-06808-w

**Published:** 2023-08-31

**Authors:** Michael Tim-yun Ong, Gene Chi-wai Man, Xin He, Mingqian Yu, Lawrence Chun-man Lau, Jihong Qiu, Qianwen Wang, Jeremy Ho-pak Liu, Ben Chi-yin Choi, Jonathan Patrick Ng, Patrick Shu-hang Yung

**Affiliations:** 1 Department of Orthopaedics and Traumatology, Faculty of Medicine, The Chinese University of Hong Kong, 74029 Hong Kong SAR, China; 2 Lui Che Woo Clinical Science Building, Prince of Wales Hospital, Shatin, Hong Kong SAR China; 3 Department of Orthopaedics and Traumatology, Prince of Wales Hospital, Hong Kong SAR, China

**Correction:* BMC Musculoskelet Disord *24**, **510 (2023)**

10.1186/s12891-023-06639-9

Following publication of the original article [1], the authors identified an error in the author name of Jeremy Ho-pak Liu. The family name “Liu” was captured as another author’s name.

The author group has been updated above and the original article [1] has been corrected.
